# ‘There is still a part of me that would love to be the old me again’, how do adolescents and young adults (AYA) experience cancer-related bodily changes: a phenomenological interview study

**DOI:** 10.1007/s11764-024-01578-3

**Published:** 2024-04-04

**Authors:** Robin T. J. Hendriks, Mies C. H. J. van Eenbergen, Marjolein L. de Boer, Sophia H. E. Sleeman, Dorry Boll, Olga Husson, Tom. I. Bootsma

**Affiliations:** 1 Department of Culture Studies, School of Humanities and Digital Sciences, Tilburg University, 5000 LE Tilburg, the Netherlands; 2 Department of Medical Oncology, Netherlands Cancer Institute-Antoni Van Leeuwenhoek, 1066 CX Amsterdam, the Netherlands; 3 Department of Research and Development, Netherlands Comprehensive Cancer Organization, 3511 DT Utrecht, the Netherlands; 4 Department of Communication and Cognition, Tilburg University, 5000 LE Tilburg, the Netherlands; 5 Dutch AYA ‘Young & Cancer’ Care Network, 3511 DT Utrecht, the Netherlands; 6 Department of Gynaecology, Catharina Hospital, Eindhoven, the Netherlands; 7 Department of Surgical Oncology, Erasmus MC Cancer Institute, Erasmus University Medical Center, 3015 GD Rotterdam, the Netherlands

**Keywords:** Cancer, Adolescents and young adults, Lived experience, Qualitative research, Interpretative phenomenological analysis, Bodily experience

## Abstract

**Purpose:**

Due to the increase in both cancer incidence and overall survival rates, more adolescents and young adults (AYAs) have to live with the effects that their cancer diagnosis and following treatments have on their bodies. This qualitative phenomenological study aimed to gain more insight into the way AYAs experience these effects and how they respond to these effects.

**Methods:**

Semi-structured interviews with a sample of 11 AYAs with an age range of 25-41 years at the time of the interview, who were diagnosed with different types of cancer, were conducted. Participants were recruited via social media and patient associations until data saturation was reached. A topic guide with open-ended questions about lived experiences was used. Interpretative phenomenological analysis (IPA) was performed to analyse the transcripts.

**Results:**

We identified six Group Experiential Themes based on different ways AYAs experience their bodies: (1) self-conscious body, (2) vulnerable body, (3) adapting to the body, (4) uncontrollable body, (5) remembering the body and (6) shared bodies.

**Conclusion:**

This study offers in-depth insight into the bodily experiences of AYAs after cancer and how they respond to these changes from a phenomenological point of view.

**Implications for Cancer Survivors:**

This knowledge could be beneficial to provide more guidance for AYAs during and after their illness, by focussing on personalised psychological (after)care.

## Introduction

In Europe alone, an estimated 120,000 adolescents and young adults (AYAs) aged 15–39 years will develop cancer [1]. In the Netherlands, there is a clear distinction between the field of paediatric oncology, which serves patients aged 0–18, and adult oncology which includes patients aged 18 and older [2]. Patients diagnosed with cancer in the 18–39 age range are often described as the ‘lost tribe’, because they fall outside the scope of specialised paediatric care yet have distinct needs that differ from older adult patients [3, 4]. On a yearly basis in the Netherlands, an estimated 3900 AYAs aged 18–39 years received their cancer diagnosis [5]. Whilst the overall survival rate has improved for both sexes, there is a considerable increase in overall cancer incidence [6], and more than 80% of AYAs are expected to survive beyond 5 years [6, 7]. As a result, AYAs must live with the long-term and late effects of their cancer diagnosis and following treatment(s) [6] such as chemotherapy, radiation and surgery, which could include amputation, hair loss, skin burns, changes in weight, limitations in physical movement [8, 9] and psychological distress[6, 8–10]. AYAs describe the experience of these changes as distressing, uncomfortable, traumatic, impactful and disruptive [9]. Therefore, cancer and treatment(s) may negatively impact important age-related milestones in AYAs’ lives, such as sexual identity, involvement with peers and dating, starting a career or employment, forming their own identity and establishing a healthy body image [11–13]. In other research for example, AYAs have referred to their changed bodies as ‘sick’, ‘ugly’ and ‘abnormal’ [14].

The growing interest in research on bodily experiences led to an increased use of quantitative data scales, also amongst AYAs, which focus on the evaluation of one’s (dis)satisfaction with their appearance [15]. An example of one of these quantitative data scales is the Body image scale (BIS), which includes a 4-point scale ranging from ‘not at all’ to ‘very much’. Questions include, for example, ‘Did you find it difficult to look at yourself naked?’ and ‘Have you been feeling feminine/masculine?’ [16, 17]. These scales provide generalizable outcomes; however, they can only measure univocal information about one’s body and cannot interpret different ways one and the same body is experienced [18]. Additionally, these scales measure aspects of how one experiences cancer on a predefined timescale [18].

A qualitative approach towards the AYAs’ agency and the surrounding context may create a more in-depth understanding about how they experience their own body [15]. Thus, to gain this in-depth understanding of the bodily experiences, a phenomenological approach was used for this study. Interview studies about bodily experiences amongst AYA survivors are scarce since they include specific patient samples with different age ranges and cultural backgrounds or only focus on a specific aspect of one’s body image [9, 19, 20]. Body image is described in literature as a dynamic, complex, multidimensional, social construct that encompasses a range of bodily experiences that could positively or negatively impact a person [9, 21, 22]. A negative body image is reported to be a long-term issue (5–20 years after diagnosis) for 15% of AYA cancer survivors, especially females [23]. The aim of a phenomenological approach lies in understanding people’s experiences and narratives [24]. Therefore, descriptions of the way one’s body and illness are lived and experienced by the person who is sick are centred [25]. From a phenomenological point of view, one’s possibilities to act in the world depend on whether one’s body appears as the focal point of one’s experiences [15, 26, 27]. The body may appear in one’s foreground because of sensations, such as pain, or due to limitations in functioning [26, 28]. These factors could lead to an impossibility to act. For example, the phenomenological philosopher Merleau–Ponty analysed cases in which the potential of the body is impaired due to brain damage, which could result in the decline of physical movement [26]. However, someone could have a physically capable body, but due to social and cultural norms, they still experience the impossibility to act [27], for example, ‘I cannot go out in public without a wig or headscarf’. Social norms and expectations around appearance can lead individuals to fear stigmatisation. This (im)possibility to act is defined as ‘I can’ and ‘I cannot’ [25, 26]. The concepts of ‘I can’ and ‘I cannot’ are used to reflect on the data [27]. These concepts combined with a phenomenological view can create a clear perspective on the way persons experience their illness and the possible disturbances it can create on their sense of self and the experienced agency [15].

To gain a broader in-depth outlook on different factors that may influence one’s perspective on one’s body, this phenomenological interview study therefore focuses on the following research questions: ‘How do AYAs experience their (changed) bodies after cancer’, and ‘How do they respond to these bodily changes?’.

## Methods

### Study population

Participants were eligible if they met the following criteria: the participant (1) received their primary cancer diagnosis between the age of 18 and 40, (2) had a life expectancy longer than one year according to the estimates of a treating physician at the time of interview, (3) were diagnosed with cancer since less than 15 years ago, (4) had sufficient Dutch language proficiency to understand and respond to interview questions.

### Recruitment

Participants were recruited via social media platforms of different Dutch cancer patient organisations and their websites (Kanker.nl, AYA-denk-mee panel, Jong Hematon, Jong Borstkanker, Stichting Jongeren en Kanker). Eleven participants agreed to participate in the interview study between April 2022 and June 2022. This sample is one above the recommended IPA sample of 4–10 participants [29]. Consecutive sampling was used since everyone who applied was eligible to participate in this study. The recruitment of participants was stopped when data saturation was attained, that is, when no new information or themes were found in the data [30] The researcher provided the participants with information about the interview study, and both verbal and written informed consent were obtained. Institutional Review Board of the Netherlands Cancer Institute declared that the Medical Research Involving Human Subjects Act (WMO) does not apply to this study (IRBd22-008/N22BIC).

### Data collection

Phenomenology was used to identify a range of dimensions of how AYAs experience their bodies after cancer [13]. The interviews were conducted face-to-face and online using Microsoft Teams [31] by the first author, who is a 29-year-old female master’s student in Health Humanities, has an educational background in applied psychology and has had training in interviewing skills. Despite the engagement with respondents, a sufficient reflexive distance from the respondents was maintained in order to conduct a thorough analysis. Upon reflection, the position of the researcher and the following perspective have been critically examined during discussions with the research team.

One participant preferred a face-to-face interview, and the preferred location was arranged [30]. The interviews were audio-recorded with a voice tracer and transcribed by the first author. Pseudonyms were given to participants in order to anonymize the data. Before the interviews took place, participants were asked to complete a short case report form (CRF) together with the researcher. The CRF included characteristics such as age, gender and specifics about the type of cancer and treatment. The interviews were semi-structured, and AYAs were first asked the general question: ‘Can you tell me a little bit about how you first found out you had cancer?’ to better understand the experience of AYAs, whilst also allowing their responses to shape the interview (Table 1 Semi-structured interview guide). Participants were encouraged to elaborate on their experiences by using prompts, such as ‘Could you give an example of that?’ The goal of the interviews was to explore changes in relation to the body, changes over time and the way participants deal with these changes.

**Table 1:** Semi-structured interview guide

Exploration of actions, situations, habits and events
**Goal: Exploration of changes surrounding the body**
How did you find out you had cancer?
What was that situation like for you?
Could you tell us a bit more about that?
Have things changed for you?
In what way has it changed?
Who was involved and how?
How did you feel in that situation?
How did you deal with that?
How do you look back on that now?
What changes have you experienced in your body?
How was that for you?
Has your environment also seen these changes?
How did they deal with that?
**Goal: Exploration of changes over time**
How did you deal with that?
What did you find difficult? And why?
How did you react?
How was that before?
What changes have you experienced in your body over time?
How do you respond to these changes now?
Are there things that you can no longer do or that you find difficult to do?
Is there anything you would like to change? And why?
Are you sometimes confronted with your changed body?
Are there things that you have received support with or would have liked to have had?
**Goal: Exploration of dealing with changes surrounding the body**
What helped you to cope?
What did you need more in hindsight? And why?
What do you find difficult about it? And why?
How did you experience that? And how do you experience that now?
What would you like to say to future AYAs?
How did you experience the conversation?
Is there anything else we haven’t discussed that you’d like to mention?
Do you have any questions for me?

### Data analysis

The six steps of the interpretative phenomenological analysis (IPA) method were used to analyse the transcripts [29, 32]. The IPA method is a qualitative analysis which puts emphasis on understanding what the participants think or believe about a certain topic by studying the meaning of experiences and events of participants [29]. Following the six steps of IPA, in the first step, the transcripts were first read and reread closely by the first reviewer to become familiar with the data. During the second step, descriptive comments were made in the transcripts with MAXQDA 2022 Analytics Pro software [33]. These descriptive comments were used to develop Experiential Statements (old term: Emergent Themes), such as ‘self-doubt’ and ‘uncertainty’ as the third step. In the fourth step, Personal Experiential Themes (old term: Superordinate Themes) were identified to look for connections between the Experiential Statements (old term: Emergent Themes). This process was repeated for each transcript as a fifth step, with the focus on the individuality of each new case. Transcripts were individually compared for patterns across cases and Group Experiential Themes (old term: Meta Themes) were identified as a sixth step. The analysis process was supervised by the second, third and last authors who have expertise in different qualitative data analysis methods in the field of psycho-oncology. Inconsistencies and questions were discussed with supervisors and solved after reaching a consensus. Group Experiential Themes (old term: Meta themes) were identified and discussed with the multidisciplinary research team, to create comprehensive topics about bodily experiences of participants. In order to ensure rigorous reporting standards for our qualitative study, we adhered to the COREQ checklist.

## Results

Table 2 (Overview of participant characteristics) shows the characteristics of the 11 participants, including eight women and three men with the age range of 25–41 years at the time of the interview. Participants in this study have been diagnosed with the following cancer types: breast cancer, thyroid cancer, testicular cancer, melanoma cancer and Hodgkin’s lymphoma. We identified six Group Experiential Themes (old term: MetaThemes): self-conscious body, vulnerable body, adapting to the body, uncontrollable body, remembering the body and shared bodies.

**Table 2:** Overview of participant characteristics

Name participant (pseudonym)	Gender	Age at time of interview	Diagnosis	Years since diagnosis	Treatment status	Type of treatment(s)
Amber	Female	38	Breast cancer	1	In treatment	Surgery Chemotherapy Radiotherapy
Bella	Female	32	Breast cancer	1	In treatment	Surgery Chemotherapy Radiotherapy
Charlotte	Female	37	Breast cancer	3	In treatment	Surgery Chemotherapy Radiotherapy Hormone therapy
Daisy	Female	36	Breast cancer	3	Finished	Surgery Chemotherapy
Emily	Female	25	Breast cancer	3	In treatment	Surgery Chemotherapy Radiotherapy Hormone therapy Immunotherapy
Faith	Female	34	Thyroid cancer	4	Finished	Surgery Radioactive iodine treatment
George	Male	41	Hodgkin’s lymphoma	5	Finished	Chemotherapy
Hunter	Male	29	Testicular cancer	8	Finished	Surgery Chemotherapy Radiotherapy
Ivy	Female	38	Breast cancer	8	In treatment	Surgery Chemotherapy Radiotherapy Hormone therapy
Jaimy	Female	28	Melanoma	10	Finished	Surgery
Kyle	Male	39	Testicular cancer	12	Finished	Surgery Chemotherapy

The self-conscious body gives an insight not only on how AYAs perceive their own body but also on how they feel about their bodies when being in the outside world, whereas the vulnerable body shows how participants experience the exposure of their bodies. Participants described a variation of physical and mental factors that they had to get used to or learn how to deal with, because of cancer, which are described in adapting to the body. The way participants experience losing control over the body and the unpredictability of cancer are described in the uncontrollable body. Remembering the body entails the experienced grief for the once capable and healthy body. The importance of sharing one’s illness experiences, which contributes to the feeling of support from peers, is included in sharing bodies.

### Self-conscious body

The process of treating cancer, inside and outside, enforced a change in how one feels about their appearance and their body. In our study, women expressed that they were generally content with how their scarring turned out, and they did however seem to experience difficulties with their changed appearance. For example, they discussed their struggles with how their breasts or scars were perceived by outsiders, especially after a mastectomy or other surgery. All six women who were treated for breast cancer felt watched and judged by outsiders, which resulted in feelings of uncomfortableness, insecurities and self-awareness. They expressed a fear of deviating from the norm or from what is seen as ‘normal’ by other people, which is, in their eyes, a woman who has two similarly sized breasts. Amber, for example, expressed the following about her experience when going to the beach:

I do have an image in my head about how you are perceived by others. Yes, yes, I do care a bit too much what others think of me, I think. (..) That and you see the scars.., so you can see the scars coming out a little bit underneath. Yes, I still have the idea that you are being watched. (Amber, 43 years old, female, breast cancer—in treatment).

Therefore, they found it important to wear a prosthesis to avoid looking asymmetrical or in their words ‘crooked’. Ivy used strategies in order to actively hide her scars from others:

I always have the idea that you can see that I am crooked.. Because I no longer have those lymph nodes, there is a dent near my armpit. That, well, you can see that in a bikini or bathing suit of course.. so then I always walk with my arm up a bit or….to disguise it a bit..(…) uncomfortable.. just.. yeah.. (..) You think that people look at you. (Ivy, 38 years old, breast cancer—in treatment).

The possibility for others to see this deviation or other differences, such as scarring, seemed to cause high levels of self-awareness of these women’s changed bodies. The men in this study, however, were less affected by the possibility for others to see their scars, as their scars were not visible. All participants did mention being afraid to be viewed as a ‘cancer patient’ by others, especially because of the loss of hair due to chemotherapy.

### Vulnerable body

Both men and women reported feelings of vulnerability and insecurity about their changed bodies, especially during intimacy. Insecurities seemed to rise and conformation and affirmation of one’s changed appearance seemed to be of importance for participants to still feel attractive, validated and accepted. Amber reported the following about her changed appearance:

He (partner) also always said: I hope you do not ever cut your hair…. Now I have short hair… he is on team long hair… me too. And now I am just different as.., it is appearance, but yes .. My appearance is just completely different from… for example what he likes, yes, he does not say anything about it, but I find it difficult. (Amber, 43 years old, female, breast cancer—in treatment).

Participants who suffered from breast cancer and testicular cancer reported feelings of insecurity or confrontation when being touched by someone other than themselves. Kyle, for example, expressed the following about being touched in his scarred area:

If it has demonstrably changed something and you can feel that too and, well you can’t see it when I am naked.. except for the scar I have on my pubic bone.. There is clearly a scar there. If someone feels that .. yes that is very different .. that is very confronting yes .. personally I am the most naked. (..) If, in a moment like that, I have to explain to my girlfriend or to a friend what happened to me. (Kyle, 39 years old, male, testicular cancer—finished treatment).

It appeared that, to avoid confrontation, women preferred to wear a bra or t-shirt during intimacy, or they avoided getting intimate with their partner. One of the reported reasons for women to cover up their bodies included that they find it difficult to see partners struggle with their changed appearance. Participants also described experiencing distance between their changed body part and their partner, especially in the frequency that the changed body part is touched compared to their other body parts. Kyle expressed the following about this:

Your own body parts get touched quicker and more easily played with than with your prosthesis. (Kyle, 39 years old, male, testicular cancer—finished treatment).

Participants expressed difficulties in discussing these matters, such as insecurities, with their partner. This seemed to lead to friction in the communication between the participant and their partner, which resulted in unclear expectations between both. Emily expressed the following about the communication between her and her partner.

I just notice that he finds that very difficult, because he knows how much pain is involved and what a uncomfortable story was behind it. He also admits that he finds that difficult, but I also find that difficult because I still want to be considered beautiful and yes I think he can just ignore his difficulties. (..) Well that he doesn’t see that scar. Yes, actually and the idea that I just have two breasts..(..) Yes I do feel insecure about it, because I do not know if he wants that, but I just do it because.. yea (Emily, 25 years old, female, breast cancer—in treatment).

In the case of Emily, this friction in communication led to the avoidance of intimacy. Participants did not only experience insecurities regarding the physical aspects of the body but also regarding its function. For example, due to chemotherapy, Kyle and Hunter became infertile. However, the insecurities that Kyle experienced were more focused on the consequences of his infertility than the infertility itself.

That also brought with it a lot of unrest for me personally, like.. what does my wife want? What does she want? Does she want a man who cannot have children? I know plenty of relationships that have broken down because of that.. because the man could not contribute to the female desire to have children. I was more afraid of the consequence that my wife would leave me. (Kyle, 39 years old, male, testicular cancer—finished treatment).

It appeared that a lack of communication between the participant and their partner generated more or new feelings of insecurity for participants. Other frequently reported experiences were the loss of sexual arousal and vaginal dryness or irritation.

### Adapting to the body

Participants discussed, regardless of the type of treatment, that they were more sensitive to stimuli, such as sound, smell and taste, and were easily tired and experienced loss of energy and muscle strength after cancer. George and Amber expressed that certain images and colours arose from physical reflexes, such as gag reflexes. George experienced nausea when exposed to the colour orange, as it reminded him of the colour of chemo. Amber experienced this when she was exposed to bright red colours, which is the same colour as ‘red devil chemo’ (doxorubicin). Participants experienced the same reflexes when looking at pictures of people who received chemotherapy. The taste and smell of metal were often associated with chemotherapy by participants. Due to chemotherapy, very strong tastes, such as sourness or bitterness, were described by participants as no longer tolerated.

Participants who had surgery also had to get used to how their body looked on the outside and had to adapt to changed sensations in the operated areas. During the interviews, participants such as Charlotte, often referred to themselves as a ‘young boy/girl’; however, their age did not seem in line with the capabilities of their bodies. Because Charlotte found this very confronting, she covered the mirrors in her house; however, she could often see her reflection in her phone.

While in your head you are still a healthy young girl. Which you aren’t... So I wasn’t looking in the mirror, but when you look at the reflection in your phone, that’s a bit of a mirror too. (…) I was often laying on the couch and I had a white pillowcase and no hair… very sick… very different than I normally looked… When I see my reflection (…) I though.. I am also a cancer patient. (Charlotte, 37 years old, breast cancer—in treatment).

Participants felt like their bodies aged fast due to cancer and treatment, as their bodies felt less capable and more fragile. It appeared that participants, who were still undergoing treatment, struggled more with adapting to their bodies than participants who already finished their treatment. Participants who were still in treatment seemed to experience more unfamiliar sensations in their bodies, such as having no sensation at all when being touched. The experience of having no feeling in certain areas of the body was often described by participants as strange or different, and a lack of sensation may cause feelings of sadness amongst participants. By touching the body and moving the body, participants appeared to be able to recognise new sensations and feelings that they were not yet familiar with. About this, Daisy reported the following:

Touching yourself and moving, that also helped me to get used to… used to how things feel.. how things move.. it all felt new. All my muscles had to learn to cope, because one of their buddies is gone. (..) Then they suddenly have to work harder and it very gradually and slowly started to feel more and more normal. Not quite yet, but for the most past yes.. What I was very surprised about at first.. about the sensation.. it is now much more in line with expectations with how it should feel, I’ll just say.. how it used to feel, it is also disappearing more. (Daisy, 36 years old, female, breast cancer—finished treatment).

Therefore, the participants’ unfamiliar sensations seemed to get more and more familiar due to time and exposure. It seemed that, when frequently touching one’s own body, sensations in the body became more in line with one’s expected sensations. Participants referred to these new sensations as the ‘new normal’ or ‘feelings of home’ when they felt more familiar with them over time. Eight participants mentioned that help from professionals such as psychologists, lifestyle coaches or pain therapists had been beneficial in learning new methods to understand and adapt to the changed body. However, it was mostly their own initiative that they came into contact with these professionals.

### Uncontrollable body

Participants expressed experiencing cancer as something that they had no control over. The feeling of having no control seemed difficult for participants to deal with. Most participants experienced fear that the cancer might return, even though their treatment was finished. Due to this fear, some participants mentioned being more aware of changes in their body and therefore also seemed to check their body more often for changes, for example, swollen lymph nodes, other bumps or pain in or on the body.

For example with my low back, I start doing exercises like an idiot to see if it goes away when I do that. And if I notice that it will not go away, then it gets in my head. And well then you feel it more often or then you think I am going to do those stretching exercises again (...) You end up in such a circle so to speak, until it suddenly disappears and then you think.. oh it was nothing after all. (Bella, 32 years old, female, breast cancer—in treatment).

Especially when appointments for check-ups were near, participants expressed feelings of stress. Participants expressed relief when receiving good news that their body is cancer-free. In comparison with other participants, Hunter expressed more feelings of acceptance towards the body regarding the possible return of his cancer. He expressed the following about this:

I can be very difficult about it, but to put very bluntly .. my body is apparently good at making a lot of extra cells, it has already done that twice now. (Hunter, 29 years old, male, testicular cancer—finished treatment).

### Remembering the body

Participants long to go back in time, before their cancer diagnosis. They express feelings of loss and seem to miss the state of how the body once was. Limitations, boundaries or pain in their body seemed to cause feelings of frustration and grief, due to the memories of how the body once functioned and how capable it was before cancer. In these moments, it seems to create a clear image for participants of what they have lost due to cancer and following treatments, as they compare the body to the ‘now’ and the ‘before’. This thinking process also becomes clear in the experiences of Daisy and Emily:

I think it is mainly the frustration.. I used to be able to do that .. and now I can not anymore…when my body hurts again or.. yes I can not do something that I could do before.. I have also lost a lot of energy. (Daisy, 36 years old, female, breast cancer—finished treatment).

I sometimes have cramps in that muscle and I think it is just… that muscle has been through a lot so I think it is just tired. And then I think oh ouch .. I do not think anyone has that cramp. Then I think.. I would not have had that cramp if I had just still had that breast. (…) And then I sometimes wonder why it had to be removed.. oh right… cancer. (Emily, 25 years old, female, breast cancer—in treatment).

Comparing the body before cancer and the body after cancer often seemed to lead to frustration and sadness. Bella who is 32 years old and is in treatment for breast cancer described this experience as a feeling as if her childhood was taken away from her. Participants also expressed the importance of the remembrance of their previous bodies. For example, Amber (38 years old) created a small statue of her breasts before she underwent a double mastectomy, and Bella (32 years old) created a scrapbook with pictures of her body before and after the mastectomy. It appeared that the way participants anticipated certain aspects of their illness might have impacted the way they remember their bodies. It seemed to help participants to create positive and meaningful memories of their bodies.

### Shared bodies

Participants expressed the importance of sharing their bodily experiences with others. They gave the impression that they preferred the possibility of sharing their bodily experiences with a peer (someone who also suffered from cancer) over friends, family or significant others. Participants expressed that the biggest difference between a peer and a loved one resides in the possibility of relating to one another. Participants discussed that they felt like peers were better able to recognise and acknowledge the impact of cancer on their bodies, which seemed to be important for participants to have their experiences justified and felt understood. Participants, such as Hunter, felt like they did not have to use as much words with peers in order to express what they experienced in their bodies and the impact that it had on their bodies.

You do not have to say as much to convey the feeling of what is going on inside you now, so to speak (…) and for the people who have not experienced it, it is a bit more difficult to understand like.. okay, what exactly is going on or what impact does it all have inside your body, so to speak…. (Hunter, 29 years old, male, testicular cancer—finished treatment).

They also discussed that people who have not suffered from cancer might find it more difficult to understand the impact of living with cancer. Even though some participants mentioned having received a lot of outside support from loved ones, they still seemed to experience feelings of loneliness when they were not able to share their bodily experiences with peers. Jaimy expressed the following about this:

And because of that I also became lonely… because of that… because yes, I can’t tell my parents everything that I would like to. And yes, you don’t get all the understanding…. Yes, they do understand for sure.. and they do have support for it but….. (Jaimy, 28 years old, female, melanoma—finished treatment).

Especially topics such as death were experienced as difficult to share with friends and family, due to the emotional load that the topic has on others. Besides sharing personal experiences, participants also shared knowledge, such as practical tips with each other, for example how to deal with nose bleeding during chemotherapy, obstipation or side effects from medication. By sharing and discussing these issues with peers, participants were able to find new ways on how to deal with complications during and after cancer.

## Discussion

By taking a phenomenological approach on bodily experiences, this study showed that cancer and its treatments affect the body and therefore also the lives of AYAs. We shed more light on the difficulties which AYAs face during and after cancer and following treatments, and how they experience these changes. The body is experienced as more sensitive, less capable, fragile, different, unpredictable, unfamiliar and ‘not normal’ in social context and in comparison with the ‘old’ precancer body. The way in which the body is experienced during and after cancer seems not in line anymore with how one’s body once functioned and/or looked. AYAs express to feel like their body aged physically due to cancer and treatments. Due to these changes, participants often seem to look back to how the body once functioned. Because of the changes AYAs experience, the focus seems to lay on the body’s incapabilities (‘I cannot’) instead of the body’s capabilities (‘I can’). As a result, participants may experience incapability (‘I cannot’) in different factors of their life. It therefore seems particularly important for AYAs to take into account and reckon with these changes of the body. Peer support plays an important role in the AYAs’ illness journey, as they can share their experiences with each other. Sharing experiences seems beneficial for the AYA to feel understood, regardless of their ages. Our research shows that cancer indeed does impact important age-related milestones of the AYAs, such as forming their own identity, developing a healthy body image or establishing relationships with others [11, 12]. The experiences and challenges that the AYAs face are described or recognised by participants regardless of their age.

Visible changes in appearance of the body, due to cancer and treatments, seem to lead to participants feeling more self-aware about their bodies. Exposing the body seems difficult for participants, especially when the body, and thus the effects of cancer and treatment(s), becomes more visible to others. Scarring, amputation and the loss of hair are aspects that were frequently reported by participants. As also reported in other studies, the increased self-awareness about their changed bodies might lead to the fear of being looked at or stared at by others [14].

Women who underwent a mastectomy reported about struggles with their changed bodies and more specifically the fear of their breasts appearing asymmetrical to others. ‘Wearing mastectomy’ or ‘going flat’ could be empowering for women, as it challenges beauty standards and shines a light on the trauma that it may cause [34]. However, women in this study and other studies [15, 35] find it difficult to resist these standards or to deviate from what is seen as normal and, as a result, prefer to wear a prosthesis instead. In terms of ‘I can’ and ‘I cannot’, in this case, a visibly changed appearance seems to lead to an attitude of incapability (‘I cannot’), as women seem to have a hard time leaving the house without a prosthesis. This incapability also seems to stem from the fear of being viewed as a ‘cancer patient’ by others, due to the visibility of one’s asymmetrical breasts or baldness because of chemotherapy.

This experience of incapability also occurs during intimacy, since participants, who suffered from breast or testicular cancer, were reported to struggle with being touched. In other research, it is stated that the role of the partner also seems to play an important part in this, because through explicit acceptance from their partners, women were able to be more sexually active and can enjoy physical contact [36]. However, in this study, some women seem to experience lack of acknowledgment and acceptance from their partners about their own bodies and therefore also may have a harder time enjoying physical contact. Especially when participants see that their partner is having a hard time with their changed body part, for example, because they find it hard to look at, it may lead to an experience of incapability (‘I cannot’). As a result, women in this study reported regularly covering up their bodies or avoiding intimacy altogether.

Feelings or sensations which are experienced in certain areas of the body could lead to more awareness about those body parts [28]. In our study, it seems to be the lack of sensation in certain areas of the body that is experienced as difficult for participants. By touching and moving the body, participants seem to become more and more familiar with these sensations. Mainly when participants are still getting used to these new sensations, they seem to struggle with being touched by someone other than themselves. This experience seems to be in line with other research, where breast cancer participants describe this familiar sensation as ‘feeling safe’ [36], whereas in our study, they describe it as ‘feelings of home’.

Fear of the cancer returning after treatment seems to play a big role in the lives of AYAs. The stress that participants experienced in this study aligns with the results from another study, where severe fear was believed to result in distress, difficulties in coping and poorer physical health [37]. Medical care after cancer seems mostly focused on information given about symptoms or new signs rather than addressing fear associated with the recurrence of cancer [38]. Because of this, the focus of participants after treatment seems to be on detecting symptoms or signs, without the ability to deal with their fears.

## Clinical implications and future research

This study offers in-depth insight into the bodily experiences of AYAs during and after their illness and how they respond to these changes, which could be helpful for the improvement of personalised (after)care. Aspects that need to be addressed more often and would be helpful for AYAs include the following: adapting to limitations, processing one’s changed appearance, getting more familiar with changed sensations, facing the perception of others on the changed body, communicating with one’s partner about the changed body and dealing with the fear of recurrence. Thus, when giving patients information about their illness, it is also important to not only include medical information but also information focused on changes, adaptation and acceptance of the body and the emotions surrounding these aspects. Referral to professionals such as psychologists, physiotherapists and pain therapists is beneficial for the AYA in the rediscovery of the changed body after cancer. It is important that AYAs are made aware of the availability of these professionals as an integrated part of holistic patient care. Interventions focused on aftercare for AYAs may be helpful in their journey of adapting to their changed bodies during and after their cancer treatment(s).

## Strengths and limitations

The phenomenological approach used in this study collected rich interview data to explore experiences from a diverse group of AYAs, including age, type of cancer and treatment. However, some limitations about the sample should be noted. First, only Dutch participants participated. Second, like other qualitative studies [9], more women than men participated. Third, many of the participants suffered from breast cancer. Although a diverse group of AYAs aged between 18 and 40 at diagnosis was included in this study, the inclusion of one or two participants below the age of 20 would have been beneficial for this study to get a better representation of the younger AYAs. Because of the limitations that are mentioned above, one should be cautious to generalise the results to other cancer patients with other ages and social, cultural and medical backgrounds.

## Conclusion

With the use of a phenomenological perspective, we investigated the lived experience of AYAs with cancer and how bodily experiences may affect their daily lives. We focused on how AYAs experience their body and how they deal with these experiences in their own ways. These effects and the emotions that AYAs experience could negatively influence their experience of agency in their daily lives, leading to the inability to act (‘I cannot’). A holistic approach to cancer care, which integrates physical, emotional, psychological aspects and social support, is crucial for addressing the multifaceted needs of AYAs.

## Data Availability

Due to the nature of this research, participants of this study did not agree for their data to be shared publicly, so supporting data is not available.
